# Projit Bihari Mukharji, *Brown Skins, White Coats: Race Science in India, 1920–66*, Chicago: University of Chicago Press, 2022, ISBN: 0226823016, 348 pp.

**DOI:** 10.1007/s10739-023-09735-7

**Published:** 2023-10-16

**Authors:** Thiago Pinto Barbosa

**Affiliations:** University of Gottingen, Gottingen, Germany

The historiography of race/racism in science has by now long simmered around the question of the racializing impact of colonialism, especially settler colonialism, in the understanding and ordering of human diversity in (post-) colonial societies. But “what happens when the white man leaves? Does he take his masks with him?” (p. 255), asks Projit Bihari Mukharji’s *Brown Skins, White Coats: Racial Science in India, 1920–66*. The book investigates the scientific work on the biological human variation among social groups in pre- and post-independence India, focusing on a rapidly changing historical moment that also witnessed an increased scientific attention to blood, in a field that comprised a myriad of practices, methods, and theories that the author has agglutinated under the label “seroanthropology.”

Any impression that the author is attempting a neat genealogy of “seroanthopology” as a discrete discipline is dissipated at last in the conclusion, when the author acknowledges that seroanthropology is a “loose designation” under which he gathered “a cluster of scientific investments in race” (p. 251). Serological techniques indeed composed the methodological repertoire of scientists working in and between different fields of research when disciplines known as such today were still being formed and institutionalized, from anthropology and its subdisciplines to human genetics and medical research. The author also connects seroanthropology to other-than-serological racial methods (for instance in the discussion of “biometric nationalism,” a term the author has previously developed in a great record of work). Notwithstanding, the focus on seroanthropology—and on genetic research through blood samples more generally—proves to be insightful: it is precisely through the resilience and slipperiness (or, as the author prefers it, *feralness*) of the book’s historical object (race in science) that a richly textured picture of the “messy and hierarchic networks” (p. 21) of science and its politics is conveyed. Thereby, the contingency and fleetingness in which these race scientists undertook efforts to try to stabilize their objects, methods, and taxonomies become evident. A “snapshotting” approach to history and the reliance on the colonial ethnology assumption of the endogamy of caste, for example, are two of the strategies of reductionist stabilization in seroanthropological research in India.

Fruitfully achieving the difficult balance between a high level of historical detail, a synoptic overview, and theoretical advancements, the book takes the reader on an analytical journey that follows key science-actors and their racializing practices, from the conceptualization of racial difference through blood groups analysis (Chap. 1) and the genetic research on religious groups (Chap. 2) to the quantifying assessments of sensitivity to a bitter taste—phenylthiocarbamide—as a marker of genetic difference (Chap. 3) and the convergence of race, risk, and disease through the study of sickle cell (Chap. 4). The following core chapters take the reader in further theorizing steps: Chap. 5 builds upon anthropologist of science Annemarie Mol’s notion of “body multiple” to think about the multiplicity of enactments of blood as a scientific object and then theorize on objectivity, contingency, and reduction(ism) in science (p. 184); Chap. 6 thinks with different instances and motivations of refusal—particularly refusal in donating blood for research—to analyse the polyphonic refutations to race science; then, Chap. 7 examines futurity in relation to race science, shedding light on eugenicist and racial scientific projections of Indian society in entanglement with nationalistic discourses.

The book is enmeshed with eight fictive letters that affectively introduce each of its chapters. Mukharji’s exercise in “critical fabulation” not only offers a captivating reading but also inspires us to push the boundaries of our scholarly genres and experiment with different narrative resources.

The references to Frantz Fanon are not only in the book’s evocative title but they open each chapter and converge in the book’s final remarks. The conclusion offers a stimulating reading of Fanon as a theorist of alienation and precursor in the criticism against geneticizing racial essentialisms. This analysis is tied up with a timely appraisal of Paul Gilroy’s “radically nonracial humanism” (p. 264). In a conclusive jump, the author elaborates the notion of “Brown planetary humanism” as generative space for utopia beyond the Black– white dichotomy (p. 265), which closes the book with an astute surprise, leaving the reader with an analytical appetite for more.

Indeed, one of the key contributions of *Brown Skins, White Coats*, both to postcolonial studies and to studies of science, lies in how it further pushes the debate on race beyond the hegemonic binaries white/Black and colonizer/colonized, by diving into the muddy, brown, and at times turbulent waters of Indian scientists’ engagements with racialization also in post-independence times. The novel focus proposed in the book is a timely move in the current moment of a virtually global mainstreaming (and, concomitantly, reductionist appropriations of) postcolonial critique. By shedding light on the forgotten histories of seroanthropology, the author joins forces with an in fact very incipient scholarship on race and racism in post-independence Indian science.^1^ Extending the argument (now widely accepted within the STS scholarship on race) that race did not cease to exist in 1945, the book asserts that, after 1947—the year of India’s independence—race “mutated, was nationalized, and, in fact, intensified” (p. 255).

Although the book reasonably focuses on scientists working in India, it avoids the pitfalls of a national framing: the author indicates the international networks in which the book’s protagonists were embedded, even if one could wish more detail in how such international discussions, especially in the 1950s and 60s, might have affected (or not) these India-based scientists. Nevertheless, the book proves that thinking closely with the Indian case is analytically insightful for myriad reasons. For one, it demonstrates that neither the US nor Europe must be the prime locus of theorizations about race or racism in science.

Another important reason for the focus on India lies in how race has been overlooked in the historiography of anthropology and other sciences in India. Given the (re) emergence of biological essentializations within nationalist discourses in India, the urgency of tackling this gap is hard to ignore. As much as it might be difficult to disentangle anti-colonial sovereignty-assertive nation-building from difference-based exclusionary nationalism (as Fanon reminds us), the book offers a path-breaking contribution to the discussion of race in post-colonial societies by shedding light on key tensions regarding belonging, exclusion, and hierarchization in the workings of race scientists—also in relation to Hindu nationalism. Two key moments in this regard are: the discussion of Indian anthropologist Irawati Karve’s “mongrel nationalism,” which is insightfully analysed in dialogue with Latin American discussions on *mestizaje* ideologies as divergence-domesticating nationalist discourses; as well as the attention to the research of other seroanthropologists on religious minorities like Muslims and Jews in the Indian subcontinent. In this way, the book offers a sharp critique of race science in its global capillarity and demonstrates the importance of continuing the discussion on the colonial and post-colonial legacies of race in science today—especially in contexts where “race” has been made invisible or erased from the social vocabulary, like in India and elsewhere, paving the way for other scholars to continue the task it delineates. 

## Footnotes

1 For example, see Barbosa, Thiago. 2022. Racializing a New Nation: German Coloniality and Anthropology in Maharashtra, India. *Perspectives on Science* 30 : 137–166. 10.1162/posc_a_00405; Ghoshal, Sayori. 2021. Race in South Asia: Colonialism, Nationalism and Modern Science. *History Compass* 19 : e12647. DOI: 10.1111/hic3.12647; Subramaniam, Banu. 2019. *Holy Science. The Biopolitics of Hindu Nationalism*. Seattle: University of Washington Press.

